# The Hygiene of Play
**Carnegie Trust Report on the Physical Welfare of Mothers and Children.* Vol. II. Dunfermline.


**Published:** 1917-08-11

**Authors:** 


### THE HYGIENE OF PLAY.*

There is no pause in the movement which is taking each department of child life in succession into the hands of State authority. Of course, the process is a perfectly logical and inevitable result of the accepted postulate that the child is the nation's most precious asset. But this treasure comes to hand as raw material. Like our gold coinage, it has to be minted and impressed with the form of the dragon-slayer before it begins its national service. Read *educated* for *minted* and you have a very pretty parallel, and parable. Nowadays we have learned to see that while the costly process is going on other forces may intervene to flaw or destroy the product during manufacture. It is of no use, as Sir George Newman has taught us, to give the choicest intellectual food to a child that is too physically ill to assimilate it. We seek for the causes which disable one-sixth of our primary school children after death has taken a heavy toll, to find ourselves surprised that so many survive the unfit conditions of housing and feeding, of the exhaustion and ignorance of their mothers, of want of sufficient sleep and healthy outdoor exercise. Upon the startled realisation of these facts there has naturally followed an outburst of effort, both amateur and official, to mend matters. The second volume of the Carnegie Trust Report shows what has been and is being done in the matter of play and its influences.

We are accustomed to be told that as a nation we have lived too much for sport and games. On the other hand, we have often lately been assured that the sporting instinct evident in our officers and latent in our men has done much for us in the war. Our own attitude of mind seems generally to be as uncertain as the criticism. On the one hand we use the expression "playing the game" as an epigram embodying our idea of rectitude in action, and if we say "It isn't cricket" we mean utter condemnation. Yet we speak slightingly of one who "merely plays at" a task, and call an easy job "child's play," as if its game were not a most serious reality to a child. And while our public schools give organised games the position that made possible the old phrase about Eton playing fields and Waterloo, we have till lately thought very little of their educative value, physical or psychological, to the child of the crowded city, who has often no playground but the motor-haunted street and no material but the things intended for other uses which he may be able to requisition. Many good people have been shocked to hear that juvenile crime has increased during the war. It is not reassuring to be told that in seventeen of our largest towns the punishable cases rose from 2,686 in the three winter months of 1914-15 to 3,596 in the corresponding period of 1915-16; roughly, one-third more. In America the educational thinker has long out-distanced us in studying the subject all round, and education authorities in the provision of safe, open spaces for summer and dry-floored and roofed winter shelters. Here "free" play may be carried on and organised games enjoyed under just so much supervision and leadership as are needed to make them a success. The great recreation-houses of Chicago, divided into floors where infants, girls, boys, lads, and grown-ups are all provided for, set us an example which but for war expenses we might now be copying. It is, we believe, claimed that at least the maintenance, if not the prime cost, of such institutions is repaid by the lessened cost of remedial measures in hospital or prison. The fence is always in the end cheaper than the ambulance.

At present in our elementary schools a game, a team race, a dancing step, skipping, or jumping are only introduced at the end of some of the usual three weekly lessons in physical exercises of twenty minutes each. Local authorities in some places have made provisions of their own for organised games. Thus, Manchester began in 1910 a scheme by which children in Standards IV. to VII. have for the five months May to September inclusive about 1¼ hours' games daily in one of the parks. The work of the Children's Happy Evenings Association in London, initiated by Miss Heather-Bigg in 1888, and of the Play Centres begun by Mrs. Humphry Ward in 1897, has included besides the indoor evening amusements some playground experiments in summer. Many evening plans both in London and the provincial towns have had to be abandoned during the last two winters on account of street dangers and the scarcity of voluntary helpers. They have, however, apart from the good to the children, given great help to teachers by showing the preferences of different ages, very useful indicators of development, and also the effect upon different natures of the discipline of a well-played game. Such excellent qualities as "alertness," fairness, courage, self-control, honesty, initiative, consideration for the weak, are encouraged in the mind, and the use of eyes, ears, and muscles in the body.

In America training courses for play leaders and teachers have long been established. At Chicago the course takes over a year, devoted to lectures and practical work. Pittsburg and Wisconsin Universities have professors of physical education and play. In England the D.Sc. degree was given last year by London University to Miss Mabel Jane Reaney for a brilliant thesis on "The Psychology of the Organised Group Game." This excellent survey of the whole subject has been reprinted by the Cambridge University Press, and will serve as a very useful text-book after the war, when the appointment of play leaders and teachers is likely to proceed rapidly. Some of the information given is in itself very curious and interesting. For instance, answers to *questionnaires* sent out to large numbers of children showed developments of taste with age—"The curve for hide and seek" in one case "rises from 5 per cent. at seven years of age to 55 per cent. at ten, and falls rapidly to about 20 per cent. at twelve, and 0 per cent. at eighteen." Another "notes a distinct lack of power of organisation among girls." Miss Reaney's own *questionnaire* showed that in boys' schools 8 per cent., but in girls' schools 24 per cent. were in favour of organisation by the teacher. It also brought a strong expression of opinion from teachers that the playing of games well proved good general intelligence, and in definite cases could be seen to develop good moral qualities.

To readers of The Hospital the subject cannot fail to be of interest, like all else that can promote mental and physical health, and they will heartily endorse Dr. Janet Campbell's conclusion that "the demand for more play is an indication of the recognition of the meaning and value of school hygiene. . . . Medical inspection, medical treatment, provision of meals, school baths, open-air education, physical training and play, all contribute towards the rearing of a happy, healthy, race of children, and the influence of play is not the least among them."

* *Carnegie Trust Report on the Physical Welfare of Mothers and Children.* Vol. II. Dunfermline.

